# Potential of an Interactive Drug Prevention Mobile Phone App (Once Upon a High): Questionnaire Study Among Students

**DOI:** 10.2196/games.9944

**Published:** 2018-12-04

**Authors:** Máté Kapitány-Fövény, Eszter Vagdalt, Zsófia Ruttkay, Róbert Urbán, Mara J Richman, Zsolt Demetrovics

**Affiliations:** 1 Department of Addiction Semmelweis University Faculty of Health Sciences Budapest Hungary; 2 Drug Outpatient Centre Nyírő Gyula National Institute of Psychiatry and Addictions Budapest Hungary; 3 Budapest Center for Vocational Education and Training in Engineering Budapest Hungary; 4 Creative Technology Lab Moholy-Nagy University of Art and Design Budapest Hungary; 5 Institute of Psychology Eötvös Loránd University Budapest Hungary

**Keywords:** secondary prevention, adolescent, mHealth, energy drinks, substance use, alcohol abuse, cannabis

## Abstract

**Background:**

In recent years, drug prevention networks and drug education programs have started using Web-based or mobile phone apps as novel prevention tools, testing their efficacy compared with face-to-face prevention.

**Objective:**

The aim of this study was to assess the potential of an interactive app called Once Upon a High (VoltEgySzer).

**Methods:**

The app approaches drug prevention from 6 different aspects, and it addresses youngsters with 6 different modules: (1) interactive comics/cartoons, telling stories of recovery; (2) quiz game; (3) roleplay game; (4) introduction of psychoactive drugs; (5) information on the somatic and psychological effects of psychoactive substances; (6) list of available treatment units, rehabs, and self-support groups in Hungary. Students of 2 vocational schools and 2 high schools filled out a questionnaire at a baseline (T0) and a 2-month follow-up (T1) data collection session. Students of 1 vocational school and 1 high school downloaded the Once Upon a High app (app group), whereas students from the other vocational school and high school did not (nonapp group). The time points of T0 and T1 questionnaires contained demographic variables, items with regard to substance use characteristics for both legal and illegal substances, including novel psychoactive substance, exercise habits, knowledge about psychoactive substances, attitudes toward substance users and validated instruments measuring the severity of tobacco (Fagerström Test for Nicotine Dependence), alcohol (Alcohol Use Disorder Identification Test), cannabis (Cannabis Abuse Screening Test), and synthetic cannabinoid consumption. Beliefs about substance use (Beliefs About Substance Abuse) and perceived self-efficacy (General Perceived Self-Efficacy) were also measured. At T1, members of the app group provided additional evaluation of the app.

**Results:**

There were 386 students who participated in the T0 session. After dropout, 246 students took part in T1 data collection procedure. Alcohol was the most frequently consumed psychoactive substance (334/364, 91.8% lifetime use), followed by tobacco (252/386, 65.3%, lifetime use) and cannabis (43/323, 13.3% lifetime use). Decreased self-efficacy (beta=−.29, *P*=.04) and increased daily physical exercise frequencies (beta=.04, *P*<.001) predicted higher frequencies of past month energy drink consumption, whereas elevated past month alcohol consumption was mainly predicted by a decrease in negative attitudes toward substance users (beta=−.13, *P*=.04) in the regression models. Once Upon a High was found to be effective only in reducing energy drink consumption (beta=−1.13, *P*=.04) after controlling for design effect, whereas perceived utility of the app showed correlation with a decreasing alcohol use (r_S(44)_=.32, *P*=.03). The roleplay module of the app was found to be the most preferred aspect of the app by the respondents.

**Conclusions:**

The Once Upon a High app can be a useful tool to assist preventive intervention programs by increasing knowledge and self-efficacy; however, its efficacy in reducing or preventing substance use needs to be improved and further studied**.** Additional potential impacts of the app need further testing.

## Introduction

### Background

The era of contemporary substance use scene is also an era in which Web-based communication influences our daily lives to an increasing extent. Spending an increasing amount of time in virtual spaces is a phenomenon that mostly affects the lifestyle of adolescents [1]. Virtual spaces—such as Web-based fora, blogs, Web markets—serve a significant role as one of the primary sources of gathering either classic or novel psychoactive substance (NPS)-related information as well. The Web-based space not only provides information and means of communication, but Web-based marketing is also responsible for increasing the availability of NPS [2].

Not only the drug market but prevention itself also transformed as one of the many consequences of the rise of information technologies. In recent years, drug prevention networks and drug education programs have started using Web-based or mobile phone apps as novel prevention tools [3]. Such virtual methods may provide an opportunity to access individuals who otherwise might not seek or receive professional help. The anonymity and accessibility of prevention apps often lead to increased self-disclosure with regard to sensitive subjects such as substance consumption [4]. The effectiveness of Web-based interventions providing personalized feedback and mobile phone apps in reducing the intake of psychoactive substances is already supported by empirical evidence [5,6]. According to the findings of previous research, such programs may result in significant decrease in alcohol consumption among heavy drinkers [7], in prolonged smoking cessation as a supplement to nicotine patch therapy [8], or in decrease of marijuana use among college students who show a high level of contemplation to be abstinent [9]. However, there are some contradictory results as well with regard to the efficacy of such programs when implemented in the adolescent population. On the basis of the systematic review of Majeed-Ariss and colleagues [10], some apps *may be considered feasible health interventions* that may increase self-management of chronic health conditions, but on the other hand, there are several apps available on the app market without any evidence-based background. A Web-based prevention program for ecstasy and NPS [11] was reported to be efficient in reducing adolescents’ intentions to use NPS; it increased knowledge about both ecstasy and NPS. However, changes in lifetime use of ecstasy or NPS did not differ significantly between the intervention and control conditions.

The result that tailored and interactive sites and apps are more effective than static ones [12] is also essential with respect to our project, although this principle applies not only to apps and websites but also to prevention itself. Interactivity may also increase the subjective feeling of self-efficacy and self-directedness, which might improve the commitment to participate in a prevention program. Self-directedness itself was found to be a relevant protective factor against opiate addiction [13], alcohol addiction [14], cannabis addiction [15], and smoking [16].

As opposed to personal, face-to-face prevention, preventive interventions implemented in the virtual space have distinct advantages as they are available any time, are more cost effective in the long run, are better in providing tailored feedback, and are able to reach more members of the target population [17]. Previous studies have also indicated that mobile phone apps combine the benefits of Web-based and computerized preventive interventions as they provide both interactive and static contents with and without an internet connection [18], even if some apps cannot operate properly offline. Nevertheless, updates concerning eventual content change are usually assured by automatic version refreshments in every case.

With respect to the methodological concept of our project, we followed the concept of gamification. Gamification—when utilized in prevention—is usually defined as the mixture of game design elements and traditional prevention techniques in a nongame context [19]. The aim of gamification is to increase the engagement and motivation of the target group while providing a useful method in supporting learning and problem solving. It is not essential to use any digital technology in gamification, but smartphones make the implementation easier and the outreach wider. With regard to the efficacy of gamification in preventing substance use among adolescents, Boendermaker and colleagues [20] found that game elements (authors label them as *serious games*) can help to motivate youngsters to do a cognitive bias modification training, which might reduce substance consumption. However, research is still lacking to draw further conclusions about the efficacy of gamification.

### Objective

The aim of this study was to assess the effectiveness of a mobile phone prevention app, titled *Once Upon a High* (the original name of the app is *VoltEgySzer* in Hungarian). We aimed to target adolescents in an age range that may indicate the existing experiences with substance use but without clinically relevant problems. Therefore, Once Upon a High was tested as a novel tool with embedded game elements (ie, as a form of gamification) for mainly the purpose of secondary prevention. Being such an instrument, we expected this app to be effective—among other goals—in reducing or at least maintaining substance use frequencies. The app, besides being a contemporary gadget of providing information on the risks of substance use, was expected to enhance or affect certain skills as well, which might be considered as protective factors against the increasing severity of substance consumption and are as follows: (1) *self-efficacy* is usually seen as a protective resource in prevention or even treatment of substance use disorders (SUD) [21] and as an important determinant of health behavior in general [22]; (2) *increasing physical exercise* is similarly recommended as a potential tool to be utilized in substance use prevention for the youth [23,24] or as a beneficial adjunctive treatment of SUD [25], which may lead to elevated abstinence rates or the ease of withdrawal symptoms [26].

In the study, besides the exploratory analysis of the app’s utility, the following hypotheses were tested:

Once Upon a High can be effective in:

H1: Increasing knowledge about the risks of psychoactive substances

H2: Enhancing perceived self-efficacy

H3: Decreasing or maintaining the frequency of current substance use

H4: Decreasing negative attitudes toward substance users

H5: Increasing the frequency of physical exercise

Although H2, H4, and H5 might also be related to primary prevention, we still considered self-efficacy, physical exercise, and negative attitudes toward substance users as potential protective factors against the exacerbation of substance use among active users; and as such, preferable outcomes of secondary prevention too, potentially leading to decreased substance use frequencies. We further hypothesized that increased knowledge (H1), perceived self-efficacy (H2), physical exercise (H5), and decreased negative attitudes toward substance users (H4) might predict decreased frequencies of substance use (H3).

## Methods

### Once Upon a High: Introducing the App

*Once Upon a High* is an interactive drug prevention app targeting the youth and drawing attention to the importance of prevention in a novel way. The app approaches drug prevention from 6 different aspects, and it addresses youngsters with 6 different modules: (1) interactive comics/cartoons, telling stories of recovery (see Animated Comics section); (2) quiz game (see Quiz Game section); (3) roleplay game (see What If? section); (4) introduction of psychoactive drugs (see Substance Store section); (5) information on the somatic and psychological effects of psychoactive substances (see Trans-formation section); (6) list of available treatment units, rehabs, and self-support groups in Hungary (see Where to go? section). The user may choose to use any of the modules from the initial menu. The app unites the experience of usual Web education and computer games. The applied genres—cartoons, videos, and animation—are anticipated to be attractive to the youth by their nature. The app provides anonymity for its users.

The app was designed and developed in a joint project of Nyírő Gyula Hospital, National Institute of Psychiatry and Addictions and the techLab of the Moholy-Nagy University of Art and Design, Budapest. The development of the app was funded from a national tender (invited by the Hungarian Government’s Ministry of Human Capacities) supporting the establishment of novel drug prevention programs. More information on the technical details are presented in the Multimedia Appendix 1 as well as on the visual rendering of the app. To increase risk perception, yet to avoid overestimation of the dangers of specific psychoactive substances (ie, avoiding autotelic deterrence), the literature basis for each module contained current papers providing high-quality evidence (ie, mainly systematic reviews and meta-analyses or randomized controlled trials). The literature basis for each module is presented under the detailed characteristics of the app.

### Detailed Characteristics and Aims of the Modules

#### Animated Comics: Interactive Recovery Stories

**Figure 1:**
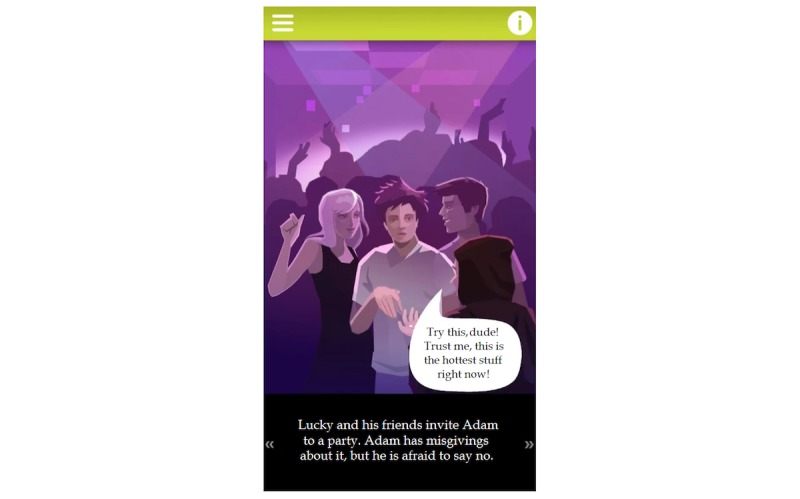
Visual appearance of the animated comics module.

The first module comprises altogether 4 recovery stories, of which there are stories of 2 males (Adam, the beggar; Adam, the prince) and 2 females (Eve, the beggar; Eve, the princess). These 4 comic-style animated tales (Figure 1) introduce common risk factors of adolescent psychoactive substance use (eg, peer pressure, peer recognition for substance use involvement, social isolation, a dysfunctional family background indicated by symptoms such as familial substance use and either sexual, physical, or emotional victimization) based on the findings of former studies [27,28], as well as potential way outs by presenting 4 types of treatments: (1) art therapy, (2) individual psychotherapy, (3) group therapy including self-support groups and as a combination with family counseling, and (4) animal-assisted therapy. The selection of these therapeutic interventions was based on former findings with respect to their efficacy in addiction treatment. Art therapy is commonly used throughout the process of detoxification and rehabilitation, including 12-step programs and self-support groups [29]. The effectiveness of individual psychotherapy and primarily cognitive behavioral therapy in the treatment of SUD was supported by the results of numerous meta-analyses [30,31]. Group counseling and family therapies were highlighted as the most effective interventions in a study comparing the effectiveness of various treatment methods for adolescent substance use [32]. A systematic review and meta-analysis of randomized controlled trials further confirmed the efficacy of animal-assisted therapy in the treatment of SUD [33]. The interactivity of this module means that the dialogues are triggered by the user, and turns are indicated by a slight movement of the main characters. The module also aims to display different socioeconomic backgrounds of substance users by telling the stories of both socially marginalized youth and youngsters coming from high-income families.

#### Quiz Game

The second module, a quiz-game, contains a database of 50 potential questions, of which the app randomly generates 15 questions every time the user plays the game. The quiz game is structured by 3 difficulty levels (easy, moderate, and hard). Difficulty levels were determined by assigning an adjustment weight to the questions based on the results of 98 high school students who gave either correct or wrong answers to all the 50 questions. Many of the quiz questions reflect common misbeliefs about the prevalence and risks of substance use (eg, by asking: “What percentage of high school students in your country have tried an illegal substance at least once in their lifetime?”). As a trend, adolescents often overestimate peer substance use [34], which might legitimize their own substance consumption.

Review of the current literature resulted in the following relevant areas, addressed in the quiz game: the topics of cannabis-induced psychosis [35], effects of energy drink consumption [36], dangers of adolescent alcohol use [37], peer group influence on adolescent smoking [38], or harms of NPS [39]. Less emphasis was placed on the risks associated with the use of substances with low prevalence among adolescents, such as heroin or other opioids, based on the findings of the most recent ESPAD study [40]. To every question, there are 3 potential answers (A, B, and C solutions), of which only one is correct. After every round, the user receives a feedback on his or her answers, which contains relevant citations from the scientific literature as well.

The quiz game has 2 main goals. First, to provide well-established knowledge of the epidemiology and risks of substance use, with respect to both legal and formerly banned “classic” substances (such as alcohol, tobacco, ecstasy, amphetamines, or cannabis) and NPS (in this module, mainly synthetic cannabinoids and synthetic cathinones). Second, the module aims to promote health-conscious behavior. Those users who are able to achieve a 100% quiz result are offered free admission to gyms, dance schools, laser-tag, and paintball facilities or indoor climbing centers. As such, this module might increase physical exercise frequencies as a potential benefit and, therefore, its application was considered to test our H5 hypothesis.

#### What If? Roleplay Game and Dialogue System

**Figure 2:**
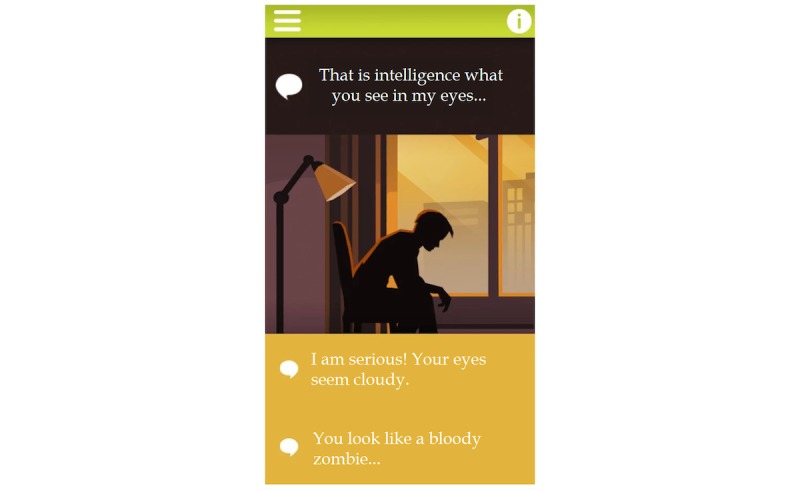
Visual appearance of the What if? roleplay game module.

The third module titled “What if?” is an interactive dialogue system, within the confines of a roleplay situation: *What if one of your close acquaintances started to use synthetic cannabinoids*? *What would you suggest*? *How would you help*? During the roleplay game, the user communicates with his or her assumed acquaintance by following a structured dialogue system, in which the virtual “talk partner” makes a specific statement, whereas the user selects a response from 2 optional sentences offered (see Figure 2). The main task within the game is to persuade the virtual acquaintance to change his lifestyle by reducing his substance use, seeking professional help, or starting drug-free recreational activities. Whenever the user selects the better option, the portrayed room gets brighter and brighter and vice versa, in case of a worse option selection, the room goes darker and darker. If the user is able to convince the virtual partner to make a positive change in his life, the game ends positively. However, in case of bad choices during the dialogue (eg, by being too impatient, hypocritical, or arrogant), the game ends with a *game over*. In this case, users receive a feedback on how to improve their communication skills and strategy next time.

The roleplay game, therefore, aims to improve communication skills, decrease stigma, and negative attitudes against people (see H4) who use psychoactive substances and to increase willingness to help peers with substance use problems. As such it targets adolescents with or without any former substance use experiences. Communication style of the virtual partner was based mainly on clinical experiences with substance users as well as the feedback of the co-design group (see Appendix for details). The assumption that peer support might be of utmost relevance was supported by literature evidence as adolescents tend to prefer informal sources of help (eg, friends) rather than professional support [41]. Finally, the risks of synthetic cannabinoid use presented in this module were also collected from published data [42].

**Figure 3:**
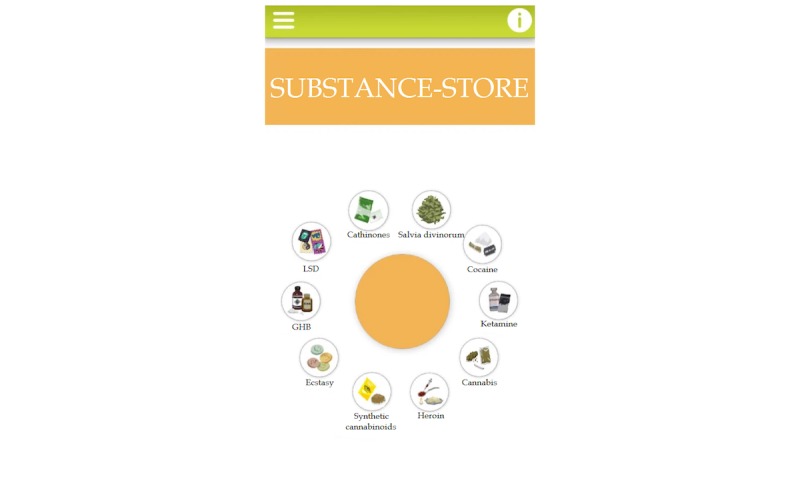
Visual appearance of the Substance-store module.

**Figure 4:**
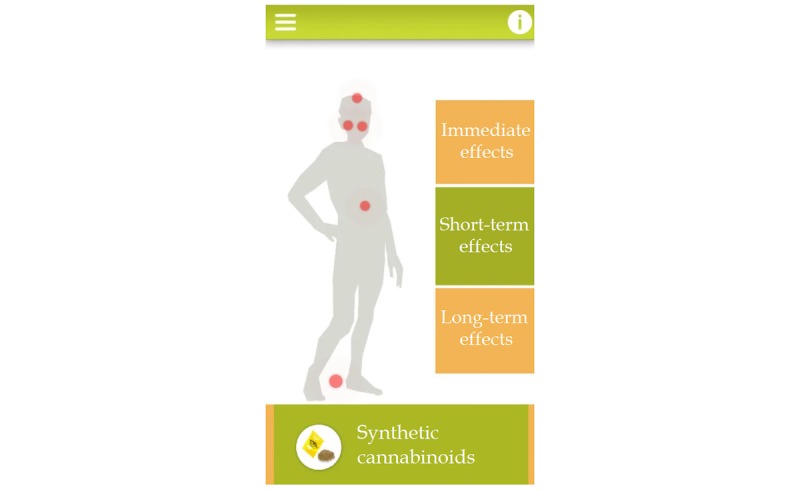
Visual appearance of the Trans-formation module.

#### Substance Store: Information About Psychoactive Drugs

The fourth module titled “Substance-store” (Figure 3) provides information on “classic” substances (cannabis, cocaine, ecstasy and amphetamines, lysergic acid diethylamide [LSD], and heroin) and NPS or recently popularized substances (ketamine, gamma-hydroxybutyrate [GHB], Salvia divinorum, synthetic cannabinoids, and cathinones). Users are offered knowledge of the brief history of the selected psychoactive substance, information on the psychoactive compounds, both desired and unwanted or adverse effects of the substance, as well as the dangers of potential overdose. For each substance, an image helps to recognize the common distribution form. As such, this module’s main goal is to educate the users and provide information. Cultural history of substance use and the desired and adverse effects of specific substances were collected from the literature [43-51].

#### Trans-Formation: Somatic and Psychological Effects of Psychoactive Substances

“Trans-formation” is closely related to the fourth module (Substance store). In addition to that one, this fifth module presents substance-specific somatic and psychological effects in a longitudinal aspect by offering a selection between immediate, short-term, and long-term effects of specific substances. As impulsivity—and especially sensation seeking—usually rise during adolescence [52], for this age group, it is thought to be more relevant to focus on the immediate and short-term consequences of substance use and not just the long-term risks of recurrent psychoactive substance consumption. All the presented somatic and psychological effects are based on the findings of former studies [42,53-55]. These effects are indicated by the red dots located on a picture of a schematic body with the option to touch a dot on the screen (see Figure 4) and, thus, access detailed description of the effects. Here too, we relied on visual clues and active exploration by the user as learning methodology.

#### Where to Go? List of Available Treatment Units

The aim of the final module was to create a national register of the available treatment units (both inpatient and outpatient), rehabs, and self-support groups. Thus, the purpose of this module is to help youngsters who are already at-risk substance users in finding nationwide professional help, as close to their place of residence as possible. By overviewing the existing national registries and the websites of self-support groups, we created a searchable map with the contact details of each of the treatment units and meeting points. As a result, altogether, more than 300 locations were registered.

### Sample and Procedure

The sample of the study consisted of 386 students of 2 vocational schools and 2 high schools from Budapest, Hungary, to analyze outcome variability by school type as well. To examine the impact of age as a covariate on the outcome measures, 9th (approximately 14- to 15-years-olds), 10th (approximately 15- to 16-years-olds), and 11th (approximately 16- to 17-years-olds) graders participated in the study from each school. The age range was 14 to 18 years. Permission from the schools was obtained as well as from the parents in cases where respondents were younger than 18 years. Written informed consents were asked in every case. Our goal was to assess participants in a natural setting, that is, in their schools, with the involvement of those professionals (teachers and school psychologists) who are involved in the everyday life of these students, to model an as realistic arrangement as possible. Randomization was performed at the school level, where participants were nested in classes. Altogether 4 groups were assessed at 2 different time points.

Students of 1 vocational (VS1) and 1 high school (HS1) downloaded the Once Upon a High app, whereas students from the other vocational (VS2) and high school (HS2) did not. Those students who downloaded the app were, therefore, part of the group hereunder referred to as “app group,” whereas those who did not download the app were members of the group referred to as “nonapp group.” We wanted to avoid assigning students to both app and nonapp groups within the same schools to prevent information sharing between study groups. Schools were selected with the help and involvement of 2 school psychologists who were also involved in monitoring the data collection procedure. Therefore, a nonprobability, convenience sampling method was used. Homeroom teachers were asked to help in organizing occasions of data collection. The app itself had an option to provide further information about its use via a website. Thus, detailed psychoeducation with respect to the use of the app was not necessary. However, homeroom teachers and those colleagues who were responsible for face-to-face data collection answered any questions that might have arisen throughout the study. We additionally provided an email address to receive and answer any incoming questions. We tried to motivate students to participate (and maintain their participation) by ensuring the opportunity of winning recreational activities (ie, free admission to gyms, dance schools, laser-tag and paintball facilities, or indoor climbing centers) in case of a flawless result in the quiz-game module.

As data were collected in 2 phases (baseline and 2-month follow-up data collection), participants received a unique identifier (UID) that helped the pairing of baseline (T0) and follow-up (T1) data. UID was generated by a similar algorithm of treatment demand indicator and consisted of the following 6 characters: (1) the third letter of the participant’s surname, (2) the second letter of the participant’s first name (3) last number of the participant’s birth month, (4) last number of the participant’s birth day, (5) third letter of the maiden surname of the participant’s mother, and (6) second letter of the maiden first name of the participant’s mother. This method was found to be more effective than allowing the students to choose a UID for themselves as they were expected to forget that during the 2 months between the 2 dates of data collection.

### Measures

The baseline and follow-up questionnaire was filled by all the participants who remained in the study. Members of the app group responded to additional questions during the follow-up measurement with respect to their experiences with the app. Participants were asked to provide an evaluation on (1) the utility and (2) subjective preference of distinct modules, as well as (3) general impression about the app using a 5-point Likert scale (from 1=not at all, to 5=absolutely). We computed 2 variables (“app preference” and “app usefulness”) as a total score of the evaluative response categories with respect to the distinct modules’ preference and the app’s perceived utility.

The baseline questionnaire contained demographic variables, questions about socioeconomic status, former experiences with other prevention programs, and the family history of either alcohol, tobacco, or illicit substance use. These items were not included in the follow-up questionnaire. Both the baseline and follow-up questionnaire comprised questions with respect to the respondents’ physical exercise and sport habits (including sport types, monthly and daily exercise frequencies, measured in minutes/day), psychoactive substance use experiences (including lifetime, last year, and last month frequencies of the consumption of both legal and illegal “classic” and NPS-type drugs). NPS-type substances included synthetic cathinones (mephedrone, methylenedioxypyrovalerone [MDPV], pentedrone), synthetic cannabinoids, and GHB, as the use of these NPS was found to be relevant among Hungarian school-aged respondents in the ESPAD study [40].

With respect to the measurement of psychoactive substance use, the questionnaire further contained validated instruments such as the 6-item Fagerström Test for Nicotine Dependence (FTND) [56,57], the 10-item Alcohol Use Disorder Identification Test (AUDIT) [58,59], and the 6-item Cannabis Abuse Screening Test (CAST) [60,61]. CAST was also used for the assessment of synthetic cannabinoid use and related problems (and referred to as sCAST). AUDIT, CAST, and sCAST were only included in the first data collection procedure as these instruments screen substance use severity at an annual rate (ie, ask questions about the past 12 months), whereas FTND measures current smoking habits. Beliefs about psychoactive substances were measured by 20-item Beliefs About Substance Abuse [62], including areas such as the ability of controlled substance use or the role of craving in relapse. Knowledge about the risks and prevalence of both legal and illegal classic and NPS-type drugs were measured by 12 items. These true/false or multiple-choice questions (eg, “What is the national prevalence of lifetime cannabis use among high school students in your country?” A=15% to 20%, B=30% to 40%, C=50%, or more) tested the knowledge that the app aimed to provide for its users. A 5-point Likert scale was used for assessing the attitudes toward substance users. Respondents had to evaluate 10 statements on substance users and addiction itself (eg, “Addiction is not a disorder, it is a lack of will-power”), rating how strongly they agree with them (from 1=Absolutely disagree, to 5=Absolutely agree). A total score of negative attitudes was computed. Finally, perceived self-efficacy was measured by the 10-item General Perceived Self-Efficacy Scale [63,64], primarily dealing with self-observed coping skills.

### Statistical Analysis

Data were analyzed by SPSS 17 (SPSS Inc) [65]. Descriptive statistics were applied to provide sample characteristics. Mean age and exercise frequencies were compared by independent sample *t* tests, perceived socioeconomic status as an ordinal variable was compared by Mann-Whitney *U* test, whereas potential differences in gender distribution and repetition of year rates were analyzed by chi-square statistics. Baseline comparison of the app versus nonapp groups, vocational versus high schools, and compliant respondents versus dropouts was implemented by using independent sample *t* tests, Mann-Whitney *U* test, chi-square test, and Fisher exact test. AUDIT, FTND, CAST, and sCAST scores were compared between the app and nonapp groups by using Mann-Whitney *U* test. Gender differences in substance use frequencies were analyzed by chi-square statistics and Fisher exact test.

To control for the design effect, pre- and postdata comparisons were performed by estimating treatment (intervention) effect with a series of linear regression analysis. Design effect was calculated by average cluster size and intracluster correlation. Changes in past month frequencies of less commonly consumed substances (including both “classic” and NPS-type stimulants and depressants) and the FTND scores at the postdata setting could not be examined by a regression analysis because of low response rates. The evaluation of the app was examined with descriptive statistics. Spearman rank correlation was applied to measure the connection between the repeated use of the distinct modules and the changes in attitudes, self-efficacy, knowledge, and exercise frequencies. The association between the app’s preference and perceived utility and substance use changes was also tested by Spearman rank correlation.

## Results

### Sample Characteristics

Altogether 386 students participated in the first data-collection (T0) session and 246 students took part in the second data collection procedure (T1). Main reasons of dropouts were lack of motivation to participate further in the study or missing identifiers to pair pre- and postdata. Tables 1 and 2 summarize detailed sample characteristics with respect to respondents with available identifiers.

With respect to both the pre-and posttest setting, the app and nonapp groups differed in age but not in gender distribution. In addition, a significant difference was found with respect to baseline exercise frequencies as members of the nonapp group showed higher means of daily exercise duration. In case of the posttest setting, this difference ceased to be significant. Vocational and high school students differed in perceived socioeconomic status as high school students reported higher living standards (mean 4.8, SD 0.9, *U*=14,517.5, *P*=.001) than students of the vocational schools (mean 4.5, SD 0.9). However, the app and nonapp groups did not show significant difference in terms of perceived socioeconomic status and rates of school year repetition because of failure (ie, poor academic performance).

Furthermore, respondents who participated in both data collection phases (ie, compliant participants) were compared with those who dropped out of the study, with regard to gender distribution, age, rates of year repetition because of academic failure, and substance use characteristics (lifetime and past month use of both legal and illegal substances, AUDIT, CAST, sCAST, and FTND scores). On the basis of this analysis, the group of students who dropped out of the study was characterized by a higher rate of male respondents (For N=384, χ^2^_1_=10.4, *P*=.001), higher mean age (mean 16.9, SD 0.9, *t*=−2.72, *P*=.007), higher rates of lifetime cannabis (For N=323, χ^2^_1_=4.4, *P*=.04), ecstasy (Fisher exact test, *P*=.006), amphetamine (Fisher exact test, *P*=.001), cocaine (Fisher exact test, *P*=.001), and LSD (Fisher exact test, *P*=.005) use; higher rates of last month cannabis (For N=323, χ^2^_1_=4.7, *P*=.03), tobacco (For N=252, χ^2^_1_=4.97, *P*=.03), LSD (Fisher exact test, *P*=.02), GHB (Fisher exact test, *P*=.02), and synthetic cannabinoid (Fisher exact test, *P*=.03) consumption. There were no differences in AUDIT (*U*=8296, *P*=.17), CAST (*U*=720.5, *P*=.92), sCAST (*U*=264.5, *P*=.31), or FTND (*U*=264.5, *P*=.09) scores, or in the rate of year repetition because of academic failure (N=385, χ^2^_1_=0.2, *P*=.70).

### Baseline Psychoactive Substance Use

The majority of the students have consumed alcohol at least once in their lifetimes (average rate=91.8%, 334/364), and there was no significant difference in the frequencies of lifetime and past month alcohol use between the subgroups (app vs nonapp groups; vocational vs high schools). However, vocational and high school students differed in the rates of lifetime and last month smoking. Although high school students showed a higher rate of lifetime smoking, last month frequencies were higher in vocational schools. Concerning illegal substances, respondents mainly had experiences with cannabis (average lifetime use rate=13.3%, 43/323), ecstasy (average lifetime use rate=3.1%, 10/319), and LSD or magic mushroom (average lifetime use rate=2.5%, 8/319). The nonapp group and participants from vocational schools showed higher rate of lifetime ecstasy consumption. In addition, lifetime use of cocaine was more frequent among the participants of the nonapp group. With respect to NPS-type substances, synthetic cannabinoids were found to be the most commonly used drugs (average lifetime use rate=6.6%, 21/319), whereas MDPV consumption only occurred among the students of vocational schools. Table 3 presents the results of the applied comparative analyses.

In case of baseline AUDIT, FTND, CAST, and sCAST total scores, there were no significant differences between the app and nonapp groups. However, students of the vocational schools showed higher total scores on both the CAST (mean 2.2, SD 4.3, *U*=532, *P*=.02) and the FTND (mean 3.3, SD 2, *U*=118, *P*<.001) compared with the CAST (mean 0.3, SD 0.7) and FTND scores (mean 1, SD 1.4) of high school students. Considering the severity of baseline substance use, 64 students (22.5%) indicated hazardous or harmful alcohol use (based on AUDIT cut-off: a score of 8 or more), 14 students (17.9%) showed moderate or severe cannabis use (based on CAST cut-off: a score of 3 or more), 3 students (5.8%) indicated moderate or severe synthetic cannabinoid use (based on CAST cut-off: a score of 3 or more), whereas 11 students (20.4%) showed risk for moderate or severe nicotine dependence (based on FTND cut-off: a score of 5 or more). With regard to potential gender differences, male students showed higher rates of lifetime ecstasy use (Fisher exact test, *P*=.04), whereas higher rates of lifetime smoking were found among female students (For N=384, χ^2^_1_=11.95, *P*<.001). In case of AUDIT, FTND, CAST, and sCAST, no gender differences occurred.

### Pre- and Postdata Comparisons

Among those participants with available and comparable pre- and postdata, potential changes in psychoactive substance use, knowledge about psychoactive substances, perceived self-efficacy, exercise frequencies, beliefs about substance use, and attitudes toward substance users were analyzed and compared between the app and nonapp group. Multimedia Appendix 2 presents pre- and posttest settings’ description with respect to the outcome measures for both the app and nonapp groups. Intervention or treatment effect was estimated with a series of linear regression analyses, while controlling for the design effect as well. Significant treatment or intervention effect was observed only in the frequency of past month energy drink consumption. Users of the app showed greater decrease in energy drink consumption after the implementation of the intervention. A trend increase in psychoactive substance–related knowledge and physical exercise frequencies could be seen in the app group but, compared with the nonapp group, these differences were not significant and could not be considered relevant.

**Table 1:** Sample characteristics and group differences at T0 participation.

Sample characteristics	App group		Nonapp group		Significance^a^ (app vs nonapp group)	Effect size, *r*
	VS1^b^	HS1^c^	VS2^d^	HS2^e^		
T0 participation, N	148	107	71	60	N/A^f^	N/A
Age, mean (SD)	16.7 (0.9)	16.5 (1)	16.9 (1)	17.2 (0.7)	*t*=−4.31; *P*<.001	.23
Male participants, n (%)	131 (89.1)	54 (50.9)	70 (98.6)	24 (40)	χ^2^_1_=0.1	.01
Repetition of year of study because of failure, n (%)	13 (8.8)	1 (0.9)	5 (7)	0 (0)	χ^2^_1_=0.5	.04
Perceived socioeconomic status, mean (SD)	4.5 (0.9)	4.9 (0.9)	4.5 (1.0)	4.6 (1.0)	*U*=15,387.5	.06
Exercise frequency (minutes/day), mean (SD)	103.9 (91.9)	92.8 (51.9)	169.5 (250.3)	82.9 (47.4)	*t*=−2.12; *P*=.40	.10

^a^Independent sample *t*-test or Mann-Whitney *U* test or χ^2^ test or Fisher exact test.

^b^VS1: vocational school 1.

^c^HS1: high school 1.

^d^VS2: vocational school 2.

^e^HS2: high school 2.

^f^N/A: not applicable.

**Table 2:** Sample characteristics and group differences at T1 participation.

Sample characteristics	App group		Nonapp group		Significance^a^ (app vs nonapp group)	Effect size, *r*
	VS1^b^	HS1^c^	VS2^d^	HS2^e^		
T1 participation, N	90	65	38	53	N/A^f^	N/A
Age, mean (SD)	16.7 (0.9)	16.3 (0.9)	16.7 (0.8)	17.1 (0.7)	*t*=−3.29; *P*=.001	.22
Male participants, n (%)	79 (87.8)	33 (50.8)	37 (97.4)	18 (34)	χ^2^_1_=3.7	.12
Repetition of year of study because of failure n (%)	9 (10)	1 (1.5)	2 (5.3)	0 (0)	*P*=.22	N/A
Perceived socioeconomic status, mean (SD)	4.4 (0.9)	5 (0.9)	4.3 (1.1)	4.6 (0.9)	*U*=6230	.10
Exercise frequency (minutes/day), mean (SD)	153.2 (208.9)	88.3 (48.5)	191.3 (257.5)	84.6 (135.8)	*t*=−0.13	.01

^a^Independent sample *t*-test or Mann-Whitney *U* test or χ^2^ test or Fisher exact test.

^b^VS1: vocational school 1.

^c^HS1: high school 1.

^d^VS2: vocational school 2.

^e^HS2: high school 2.

^f^N/A: not applicable.

**Table 3:** Psychoactive substance use characteristics at baseline measurement.

Category, substance, and characteristics			App group		Nonapp group		Significance^a^ (app vs nonapp group)	Effect size, *r*
			VS1^b^	HS1^c^	VS2^d^	HS2^e^		
**Legal substances, n (%)**								
	**Alcohol**							
		Lifetime use	123 (92.5)	94 (88.7)	60 (92.3)	57 (95)	χ^2^_1_=0.9	.05
		Last month	65 (56)	53 (50.5)	33 (55.9)	40 (67.8)	χ^2^_1_=2.2	.08
	**Tobacco**							
		Lifetime use	77 (52)	84 (77.6)	42 (59.2)	50 (83.3)	χ^2^_1_=2.1	.07
		Last month use	35 (45.5)	20 (24.1)	16 (38.1)	12 (24)	χ^2^_1_=0.4	.04
**Illegal substances, n (%)**								
	**Cannabis**							
		Lifetime use	14 (13.1)	14 (14.1)	10 (17.2)	5 (8.5)	χ^2^_1_=0.0	.01
		Last month use	2 (1.9)	7 (7.1)	4 (6.9)	1 (1.7)	χ^2^_1_=0.0	.001
	**Ecstasy**							
		Lifetime use	3 (2.8)	0 (0)	6 (10.3)	1 (1.7)	*P*=.04^f^	N/A^g^
		Last month use	1 (0.9)	0 (0)	2 (3.4)	0 (0)	*P*=.56	N/A
	**Amphetamines**							
		Lifetime use	2 (1.9)	1 (1)	3 (5.2)	1 (1.7)	*P*=.26	N/A
		Last month use	1 (0.9)	0 (0)	2 (3.4)	0 (0)	*P*=.56	N/A
	**Cocaine**							
		Lifetime use	1 (0.9)	0 (0)	5 (8.6)	1 (1.7)	*P*=.01^h^	N/A
		Last month use	1 (0.9)	0 (0)	2 (3.4)	0 (0)	*P*=.56	N/A
	**LSD^i^/Magic mushroom**							
		Lifetime use	3 (2.8)	1 (1)	4 (6.9)	0 (0)	*P*=.47	N/A
		Last month use	2 (1.9)	0 (0)	2 (3.4)	0 (0)	*P*=.63	N/A
	**Heroin**							
		Lifetime use	1 (0.9)	0 (0)	2 (3.4)	0 (0)	*P*=.56	N/A
		Last month use	1 (0.9)	0 (0)	2 3.4)	0 (0)	*P*=.56	N/A
**NPS^j^-type substances, n (%)**								
	**GHB^k^**							
		Lifetime use	2 (1.9)	0 (0)	3 (5.2)	0 (0)	*P*=.36	N/A
		Last month use	1 (0.9)	0 (0)	3 (5.2)	0 (0)	*P*=.14	N/A
	**Mephedrone**							
		Lifetime use	1 (0.9)	0 (0)	2 (3.4)	0 (0)	*P*=.56	N/A
		Last month use	1 (0.9)	0 (0)	2 (3.4)	0 (0)	*P*=.56	N/A
	**Pentedrone**							
		Lifetime use	2 (1.9)	0 (0)	3 (5.3)	0 (0)	*P*=.36	N/A
		Last month use	1 (0.9)	0 (0)	2 (3.5)	0 (0)	*P*=.30	N/A
	**MDPV^l^**							
		Lifetime use	5 (4.7)	0 (0)	3 (5.2)	0 (0)	*P*=.99	N/A
		Last month use	1 (0.9)	0 (0)	2 (3.4)	0 (0)	*P*=.56	N/A
	**Synthetic cannabinoids**							
		Lifetime use	6 (5.7)	4 (4.2)	8 (13.8)	3 (5.1)	*P*=.16	N/A
		Last month use	1 (0.9)	1 (1)	4 (6.9)	0 (0)	*P*=.19	N/A

^a^χ^2^ test or Fisher exact test.

^b^VS1: vocational school 1.

^c^HS1: high school 1.

^d^VS2: vocational school 2.

^e^HS2: high school 2.

^f^*P*<.05.

^g^N/A: not applicable.

^h^*P*<.01.

^i^LSD: lysergic acid diethylamide.

^j^NPS: novel psychoactive substance.

^k^GHB: gamma-hydroxybutyrate.

^l^MDPV: methylenedioxypyrovalerone.

### Explaining Changes in Past Month Substance Use

As secondary outcome measures, changes in past month frequencies of psychoactive substance use were examined using the changes in beliefs about substance use, psychoactive substance–related knowledge, perceived self-efficacy, exercise frequencies, and attitudes toward substance users as explanatory variables, applying linear regression models. An increase in past month alcohol consumption was explained by a decrease in negative attitudes toward substance users (beta=−.13, *P*=.04). Changes in past month tranquillizer misuse or overuse, cannabis use, and FTND scores were not explained by any of the predictor variables. An increase in past month energy drink consumption was explained by an increase in exercise frequency (beta=.04, *P*<.001) and a decrease in perceived self-efficacy (beta=−.29, *P*=.04).

### Evaluation of the App

Tables 4 and 5 present the results of the app’s evaluation as provided by the respondents of the app group.

Respondents highlighted the overall utility of the app and the majority of the participants thought that the app might be an effective tool in providing preventive knowledge. Fewer respondents considered the app to be a useful instrument in decreasing substance use or promoting exercise. When assessing the connection between the use of the app’s distinct modules and the main outcome measures, results of the Spearman rank correlation analysis indicated that of the 6 modules of the app, repeated use of the “What if?” roleplay module showed a significant correlation with an increase in psychoactive substance–related knowledge (*r*_S(35)_=.39, *P*=.02). A significant correlation was also observed between a decrease in negative attitudes toward substance users and the repeated use of the “Where to go?” module (*r*_S(44)_=.31, *P*=.04). Finally, a significant correlation was found between the app’s perceived general usefulness and a decreasing past month alcohol consumption (*r*_S(44)_=.32, *P*=.03), indicating that those respondents in the app group who found the app useful, showed lower frequencies of alcohol intake at the time of postdata collection.

**Table 4:** Evaluation of the app’s distinct modules by the respondents.

Modules	Utility, mean (SD)	Subjective preference, mean (SD)
Animated comics	3.3 (1.3)	3.5 (1.4)
Quiz game	3.9 (1.2)	3.8 (1.3)
What if?	3.8 (1.3)	3.9 (1.3)
Substance store	3.9 (1.3)	3.8 (1.3)
Trans-formation	3.9 (1.3)	3.7 (1.3)
Where to go?	3.5 (1.4)	3.3 (1.5)

**Table 5:** General evaluation of the app by the respondents.

General evaluation of the app	Mean (SD)
“Utilization of the app is easy and clear.”	4 (1.2)
“By using the app, I received novel and relevant information.”	3.9 (1.2)
“Visual realization fits the topic of substance use.”	3.6 (1.4)
“I think that the use of this app can change the user’s attitudes toward both legal and illegal substances.”	3.6 (1.4)
“The app is intriguing and entertaining.”	3.5 (1.3)
“The structure of the app is diverse and captures the user’s attention.”	3.4 (1.3)
“I think that the use of the app might facilitate treatment seeking among substance users.”	3.3 (1.3)
“The quality of visual realization is high.”	3.3 (1.3)
“I think that the use of this app helps in decreasing the frequency of substance use.”	3.3 (1.4)
“The app helps in the destigmatization of substance users.”	3.2 (1.4)
“The app helps in the promotion of exercise.”	3.1 (1.4)
“The app increases empathy and tolerance.”	3.1 (1.4)

## Discussion

### Principal Findings

This pilot study identified some potential benefits of the Once Upon a High app and also a considerable amount of flaws that need amendments to further increase the utility of the intervention. Our result that alcohol was the most commonly consumed psychoactive substance is in line with the results of epidemiological studies with respect to the substance use characteristics of the adolescent population [40]. Although we did not assess a representative sample, average lifetime use rate of NPS-type drugs was also comparable with the findings of the ESPAD study. Synthetic cannabinoids were the most popular NPS, especially in one of the vocational schools (VS2). Students of the vocational schools showed higher frequencies of tobacco, ecstasy, and MDPV use as well as higher scores on the CAST and FTND, indicating more severe problems associated with their substance use. As O’Malley and colleagues [66] emphasize in their study, school types and related socioeconomic status may have a significant impact on the students’ psychoactive substance use frequencies. Considering the assessed sample, vocational students indeed reported lower living standards than high school students.

With regard to the secondary outcome measures of the regression models, results indicate that a decrease in negative attitudes toward substance users might be a risk factor for an increasing past month alcohol consumption. This finding suggests that the subgoal of the app to destigmatize the population of substance users (those who consume either legal or illegal substances) might miss the aim: instead of facilitating social tolerance, it may indirectly promote an accepting attitude toward substance use itself. According to Hohman and colleagues [67], attitudes toward psychoactive substances—especially ambivalent attitudes—can affect substance use behavior. An increase in past month energy drink consumption was associated with higher frequencies of exercise. This result is in line with the findings of Larson and colleagues [68], namely, that pursuing sports or energy drink consumption is often related to higher rates of participation in organized sport activities among adolescents; thus, exercise is not exclusively a protective factor with respect to any substance use.

On the basis of both the respondents’ evaluation on the utility of the distinct modules and the pre-and postcomparative results of our pilot study, the Once Upon a High app might be overall ineffective in decreasing psychoactive substance use (with the exception of energy drink consumption), increasing exercise frequencies, or enhancing self-efficacy at least under the conditions of this study. When we, however, assessed repeated use of distinct modules of the app (and not the app in general), the What if? module showed significant correlation with an increase in substance-related knowledge, whereas the use of the Where to go? module was associated with decreased negative attitudes toward substance users. The What if? module was also one of the most preferred parts of the app by the responding students. As the “What if?” module contains gaming elements, the fact that it was found to be the most effective and likeable part of the app, once again highlights the benefits of gamification. Johnson and colleagues [69] also conclude that as a result of their systematic review, gamification can have a positive impact on health behaviors, although current literature lacks efficacy studies with respect to the effects of gamification on substance use. Nevertheless, Fleming and colleagues [70] also emphasize that gamification might have considerable potential for increasing the efficacy of mental health–related Web-based interventions.

In terms of hypothesis testing, our study could only partially confirm the H3 hypothesis, as the app appeared to be effective in decreasing energy drink consumption but not in reducing other substance use frequencies. Considering the emerging concerns about the harmful effects of energy drinks with high content of caffeine, sugar, or other ingredients (eg taurine or guarana) and the increasing prevalence of energy drink consumption among adolescents [71], this result might still be relevant. A correlation between the app’s perceived usefulness and a decreasing frequency of past month alcohol use was also found, indicating a more motivated subgroup of respondents who not just downloaded the app but also found it a utilizable tool. It further needs to be addressed that the majority of the respondents did not show severe (or even moderate) substance use problems as indicated by the cut-off scores of AUDIT, CAST, sCAST, and FTND. Furthermore, those students who dropped out from the study, showed higher frequencies of lifetime and past month substance use. Therefore, it is likely that the app could not exert its preventive effects on those students who would have needed it the most. This high-risk subgroup mainly consisted of males and older students. Assessing a sample consisting of adolescents with problematic substance use might better fit the scope of secondary prevention. On the basis of the characteristics of our sample (ie, the majority did not show at risk or hazardous substance use), it was not entirely possible to draw valid consequences in terms of the app’s secondary prevention efficacy. Although a significant increase in substance use was not found among the app users, the possibility of iatrogenic harms cannot be neglected in case of the app, as it was already noted in case of other prevention programs as well [72,73]. As Dishion and colleagues [72] pointed out, such harms might be dependent on many factors, including the adolescent target group’s homogeneity (eg, containing only at-risk individuals) or the specific phase of adolescence (eg, early vs late adolescence) at the time of the intervention.

### Limitations

Our pilot study is not without limitations. We did not use a randomized controlled design. The use of the app was not monitored coherently, even if respondents had to provide answers about their experiences with the app. The sample size for the applied postanalyses—because of the relatively high dropout rate—might be considered to be small for reliable multivariate analysis. Furthermore, a nonprobability, convenience sampling method was used that doubtlessly reduced the external validity of the study. The short period of follow-up time between T0 and T1 occasions (ie, 2 months) could also encumber the valid measurement of change, particularly in terms of substance use. Behavioral and cognitive change can be perceived as the result of a complex process with many unassessed factors in the background, including, but not limited to, family dynamics, peer pressure, other sources of psychoactive substance–related information (eg, internet fora), or motivation. For similar reasons, full causality cannot be assumed in our results.

These first experiences with the Once Upon a High app highlight the relevance of the target population’s motivation or the lack of it. We tried to motivate participants by offering them free recreational activities; however, this strategy failed to fulfill its goals. The offered possibility of costless admission to gyms, paintball facilities, or indoor climbing centers, did not show association with an increased frequency of daily exercise, and the dropout rates were still high. Therefore, other strategies need to be tested in the future with respect to their efficacy in motivating adolescents to maintain the use of this or a similar app. As adolescents susceptibly react to peer opinion and judgments, effective dissemination of such an app might be crucial as it may form its perception in the target population. If prevention apps can be presented as exciting or even fashionable tools that promote and help the self-management of various health conditions, the motivation of adolescents to use and share these might increase. As far as we are concerned, tools of extrinsic rewards (eg, money or presents) do not come into question in case of such prevention programs. On the basis of the findings of El-Hilly and colleagues [74], it is intrinsic motivation that may function as a positive drive to engagement in a prevention program and to a positive health behavior as well, whereas external social influences might rather have a negative impact on the overall efficacy of gamification. Similar results were published by Habgood and Ainsworth [75], indicating that in-game intrinsic motivators are more effective than extrinsic ones in case of educational games.

### Conclusions

To conclude, Once Upon a High app can be a useful and feasible tool to assist effective preventive intervention programs, as it was originally developed for such purposes as well, not necessarily as a stand-alone prevention instrument. It is likely that the beneficial effects of the app can be maximized when its use is supplemented with personal discussion. As Majeed-Ariss and colleagues [10] emphasize, similar apps that support personal self-management of chronic health conditions are usually implemented with the involvement of health professionals. School psychologists, health visitors, or social workers can also be involved in such projects to improve the effects of the app as well as to increase the target population’s commitment to use it.

Further research is needed to assess additional potentials of the app, and it is also necessary to find better ways to motivate the target population to repeatedly use this tool. Preventive effects of the app on substance use itself also need to be explored by using probability sampling and larger effect sizes and increasing the heterogeneity (eg, cross-cultural differences) to find established ways to improve the app so that it can be really called and function as a “prevention” app in the end. This pilot study might function as a basis for future research with such purposes.

## Appendix

Multimedia Appendix 1 Technical details of development.

Multimedia Appendix 2 Pre-and posttest description in point of the outcome measures.

## Abbreviations

AUDIT: Alcohol Use Disorder Identification Test
CAST: Cannabis Abuse Screening Test
FTND: Fagerström Test for Nicotine Dependence
GHB: gamma-hydroxybutyrate
LSD: lysergic acid diethylamide
MDPV: methylenedioxypyrovalerone
NPS: novel psychoactive substance
sCAST: Cannabis Abuse Screening Test adapted for synthetic cannabinoids
SUD: substance use disorders
UID: unique identifier
