# Supplementary material for: Potential of an Interactive Drug Prevention Mobile Phone App (Once Upon a High): Questionnaire Study Among Students
Source: JMIR Serious Games. 2018 Dec 4;6(4):e19. doi: 10.2196/games.9944 (PMC6299233; doi:10.2196/games.9944)
Supplement: Multimedia Appendix 1 [file games_v6i4e19_app1.pdf]

Appendix

Technical details of development

In the interdisciplinary team, experts from the hospital (psychologist, psychiatrist, social worker) were responsible for the general concept and the professional content, while TechLab provided expertise for the game and interaction design, visuals and programming. The design and development took place in a cyclic way, where designs for each module were „tested” by the peer users group (in addition to the medical expert), and their findings were taken into account for the final version. The overall process of development took approximately 12 months, for 1 programmer and 1 graphic designer. The app was developed by using Unity framework, to assure handling of the visual and animated content in distinct modules. Background tasks were programmed in C#. The dynamically generated movements and interactive visuals require substantial processing power, thus the capacity of (average, not the latest) smart phone had to be taken into account when designing the scenes and effects. The app was programmed to be responsive, meaning that the visual and textual content is well arranged on different screens. In this way the app can be used on tablets and mobiles, independent of screen size and resolution. The app is available for iPhones and Android tablets and phones.

Once Upon a High was in addition developed by establishing a co-design group, which means that the development of the various modules was implemented with the participation of a focus group representing the would-be users. Six high-school students were involved in the project for this reason. The peers were asked to comment on the general concept at the very beginning, the visual style of the cartoon and the look of the individual characters, the stories (credibility, familiarity), the language usage and finally, the interface and technical usage of the app. With adapting such a participatory design methodology – which is nowadays widely practiced for all kinds of design tasks – we wished to achieve that the app meets the expectations and communication habits of the potential users.

App visuals as presented by distinct modules

Figure 1: Visual appearance of the Animated comics module

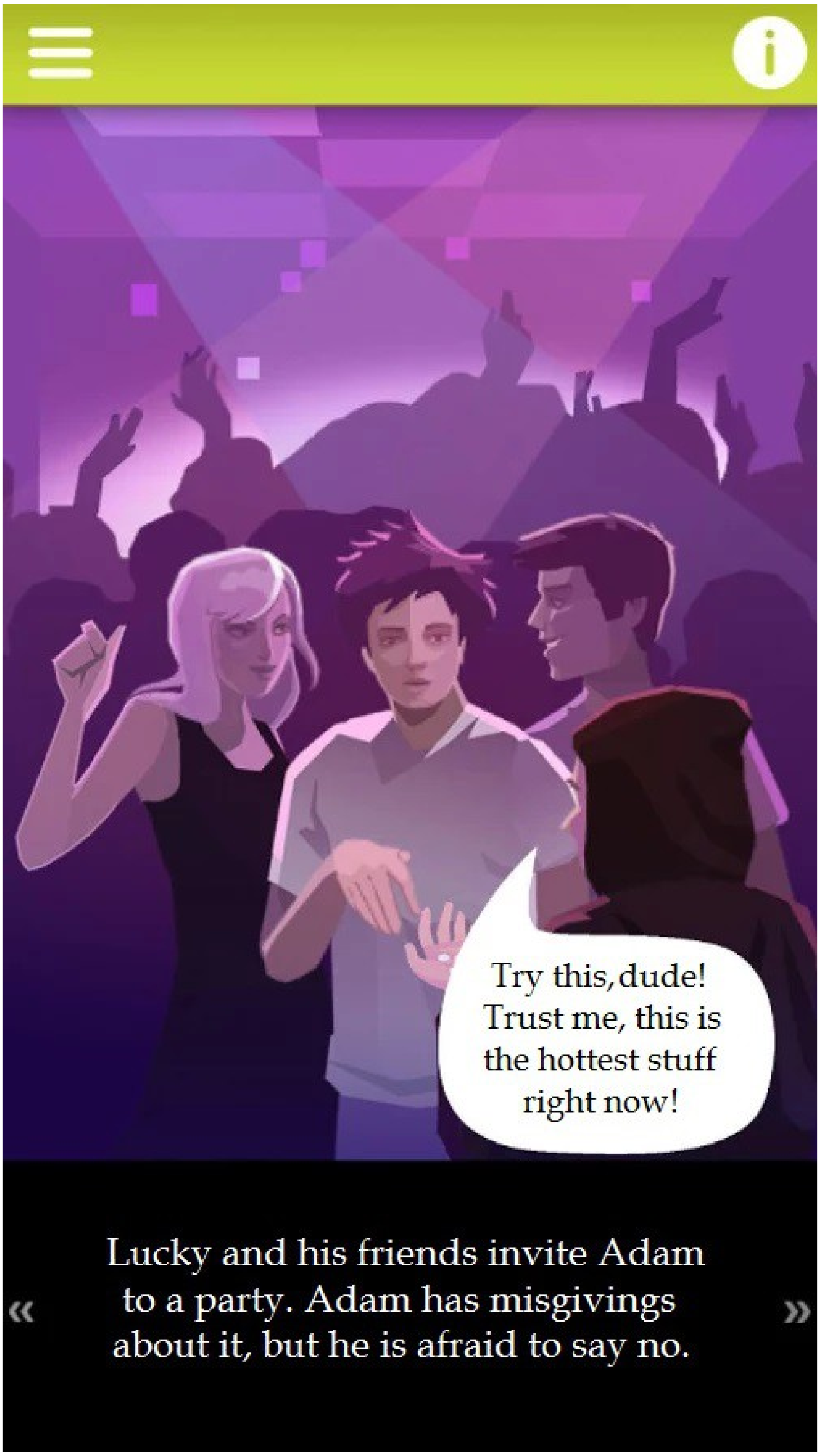
Try this, dude! Trust me, this is the hottest stuff right now!
Lucky and his friends invite Adam to a party. Adam has misgivings about it, but he is afraid to say no.

Figure 2: Visual appearance of the What if? roleplay game module

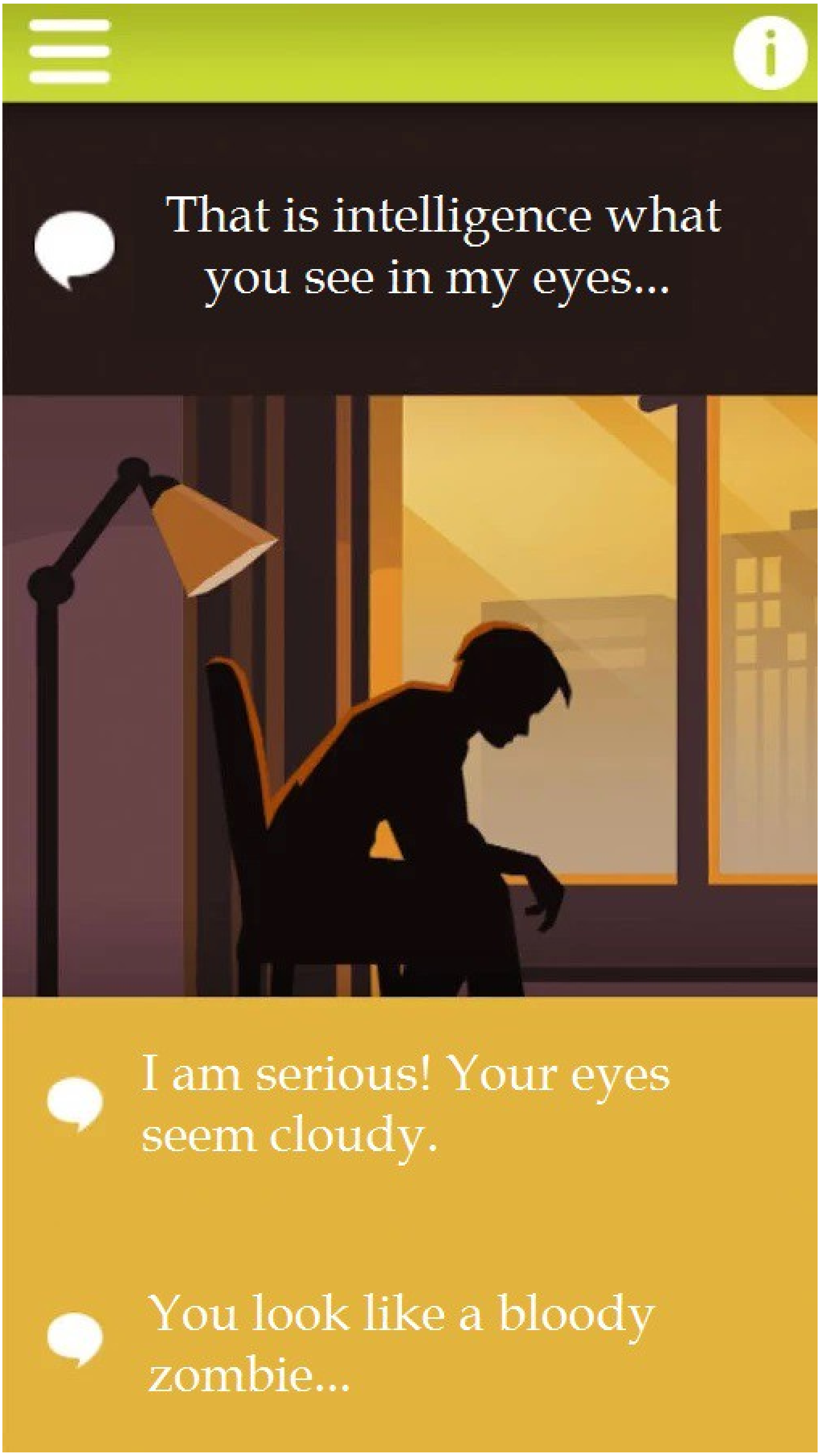

Figure 3: Visual appearance of the Substance-store module

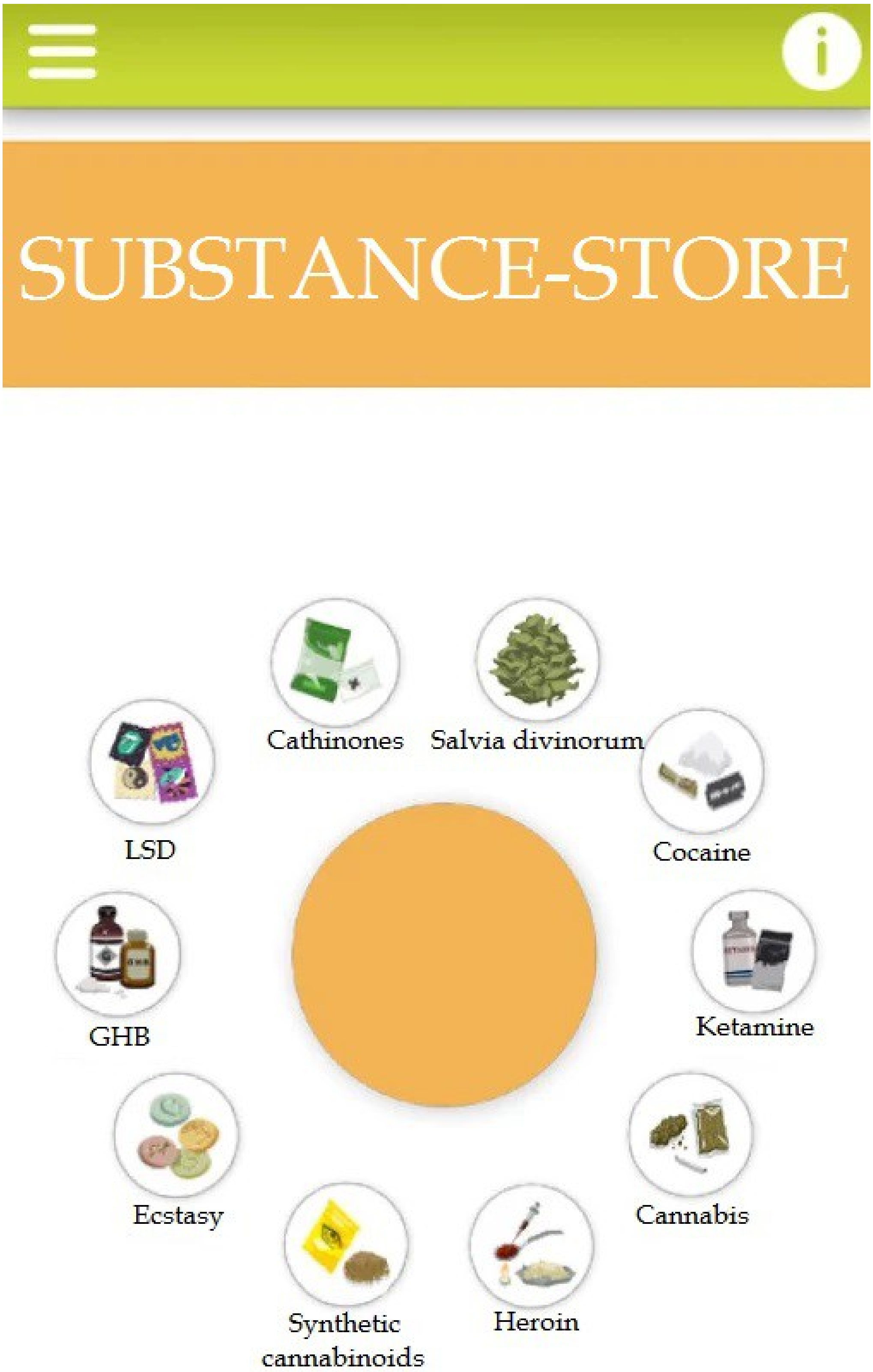

Figure 4: Visual appearance of the Trans-formation module

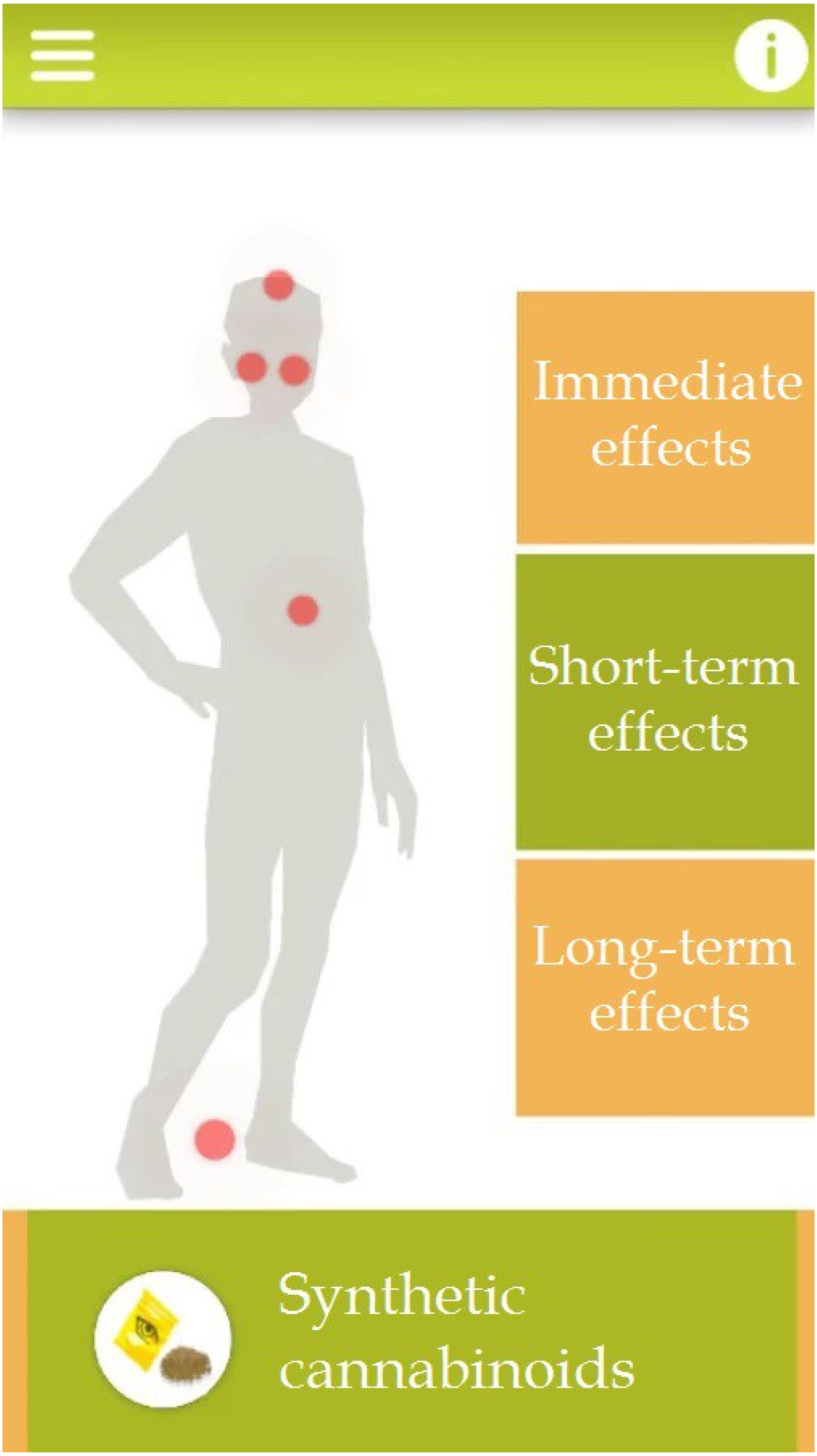
Immediate effects
Short-term effects
Long-term effects
Synthetic cannabinoids
