# Supplementary material for: Potential of an Interactive Drug Prevention Mobile Phone App (Once Upon a High): Questionnaire Study Among Students
Source: JMIR Serious Games. 2018 Dec 4;6(4):e19. doi: 10.2196/games.9944 (PMC6299233; doi:10.2196/games.9944)
Supplement: Multimedia Appendix 2 [file games_v6i4e19_app2.pdf]

Pre-and posttest description in point of the outcome measures. Treatment effect is estimated with linear regression analysis. Intervention group is coded 1 and control group is coded 0.

	App group		Nonapp group		Cluster effect		Changes[c]		Treatment effect, B (SE[d])[e]
	Pretest value, mean (SD[a])	Posttest value, mean (SD)	Pretest value, mean (SD)	Posttest value, mean (SD)	Intracluster correlation, post-pre difference	Deff[b]	App group, mean difference (SD)	Nonapp group, mean difference (SD)	
Psychoactive substance use									
Past month alcohol use	2.4 (4.7)	4 (6.9)	2.3 (3.9)	2.9 (5)	.014	1.74	-1.81 (4.95)	-0.64 (4.63)	-1.17 (0.69)
Past month energy drink use	6.2 (9.9)	6.2 (11.2)	5.6 (9.1)	4 (7.1)	<.001	1.0	0.37 (8.17)	1.49 (7.88)	-1.13 (0.46)*
Past month tranquillizer misuse	0.3 (1.1)	0.3 (1.6)	0 (0)	0.7 (4.9)	.009	1.22	-0.17 (1.46)	-1.10 (6.34)	0.93 (1.12)
Past month cannabis use	1.5 (0.1)	1.5 (0.1)	0 (0)	0 (0.3)	.010	1.26	0.00 (0.59)	-0.07 (0.32)	0.07 (0.11)
Past month synthetic cannabinoid use	1.5 (0)	0 (0)	0 (0)	0.6 (5)	.065	2.58	0.02 (0.19)	-1.00 (6.32)	-1.02 (1.23)
Knowledge about psychoactive	6.6 (1.5)	7.3 (1.8)	6.8 (1.4)	6.9 (1.8)	.035	2.51	-0.77 (1.85)	-0.25 (1.68)	-0.53 (0.39)

substances									
Perceived self-efficacy	30.9 (5.6)	30.9 (6.2)	31.5 (4.5)	30.5 (7.5)	.036	2.91	-0.24 (6.15)	1.05 (7.49)	1.28 (1.55)
Physical exercise frequencies	106.5 (89.7)	124.6 (162.4)	132.7 (202.1)	127.8 (200.1)	.015	1.81	-14.39 (129.2)	20.23 (193.0)	-34.62 (28.87)
Beliefs about substance use	25.1 (11)	24.9 (9.4)	24.1 (6)	26.9 (17.9)	.022	2.06	-0.17 (11.55)	-3.21 (17.96)	3.04 (3.23)
Negative attitudes toward substance users	29.8 (6.7)	28.3 (7.7)	31.3 (6.7)	28.9 (7.9)	.057	3.81	1.58 (7.17)	2.32 (10.23)	-0.74 (2.68)

Note: [a]SD: Standard error: [b]Deff: design effect is calculated with the formula: $deff=1+(m-1)\rho$, where m=average cluster size and ρ=intracluster correlation. [c]Mean difference=pre-test value - post-test value. [e]SE:Standard error: [f]the cluster effect is controlled in the SE.. $^{*}P<.05$; $^{**}P<.01$
